# Unraveling ionic switching dynamics in high-*k* dielectric double-gate transistors via low-frequency noise spectroscopy

**DOI:** 10.1186/s40580-025-00512-2

**Published:** 2025-10-03

**Authors:** Soi Jeong, Chang-Hyeon Han, Been Kwak, Ryun-Han Koo, Youngchan Cho, Jangsaeng Kim, Jong-Ho Lee, Daewoong Kwon, Wonjun Shin

**Affiliations:** 1 Department of AI Semiconductor Engineering, Hanyang University, Seoul, 04763 Republic of Korea; 2 Department of Electrical Engineering, Hanyang University, Seoul, 04763 Republic of Korea; 3 Department of Electrical and Computer Engineering and Inter-University Semiconductor Research Center, Seoul National University, Seoul, 08826 Republic of Korea; 4 Department of Semiconductor Convergence Engineering, Sungkyunkwan University, Suwon, 16419 Republic of Korea; 5 Department of Electronic Engineering, Sogang University, Seoul, 04107 Republic of Korea; 6 Department of System Semiconductor Engineering, Sogang University, Seoul, 04107 Republic of Korea; 7 Department of Electrical and Computer Engineering, Seoul National University, Seoul, 08826 Republic of Korea

## Abstract

**Abstract:**

High-k dielectric materials such as HfO_2_ have garnered significant attention for their potential applications in advanced electronic devices due to their superior dielectric properties. Particularly, oxygen vacancies within these materials can be strategically utilized to implement memory functionalities. However, the precise analysis of the electrical, chemical, and electrochemical characteristics related to oxygen vacancies remains challenging. In this study, we fabricated a double-gate thin-film transistor (TFT) structure employing HfO_2_ as the gate dielectric for both top and bottom gates, with the oxygen vacancy concentration intentionally modulated by introducing a TiO_2_ interlayer at the bottom gate stack. This TiO_2_ layer effectively increases the oxygen vacancy content within the bottom gate dielectric, facilitating oxygen vacancy migration-based memory operation primarily through the bottom gate. The resulting asymmetry between the top and bottom gates was systematically analyzed using low-frequency noise (LFN) characterization, elucidating for the first time the distinct impacts of oxygen vacancy modulation on device electrical behavior and operational mechanisms. This comprehensive LFN analysis provides critical insights into the fundamental dynamics of defect-mediated memory operation, highlighting the importance of dielectric engineering in optimizing next-generation oxide-based electronic devices.

**Graphical abstract:**

This study unravels ionic switching dynamics in double-gate HfO2–IGZO TFTs, where a TiO2 scavenging layer modulates oxygen vacancies to enable memory operation. Low-frequency noise spectroscopy reveals a ionic-dependent transition between distinct noise mechanisms, providing fundamental insights into vacancy-driven dynamics and guiding the optimization of high-k dielectric transistors for next-generation computing.

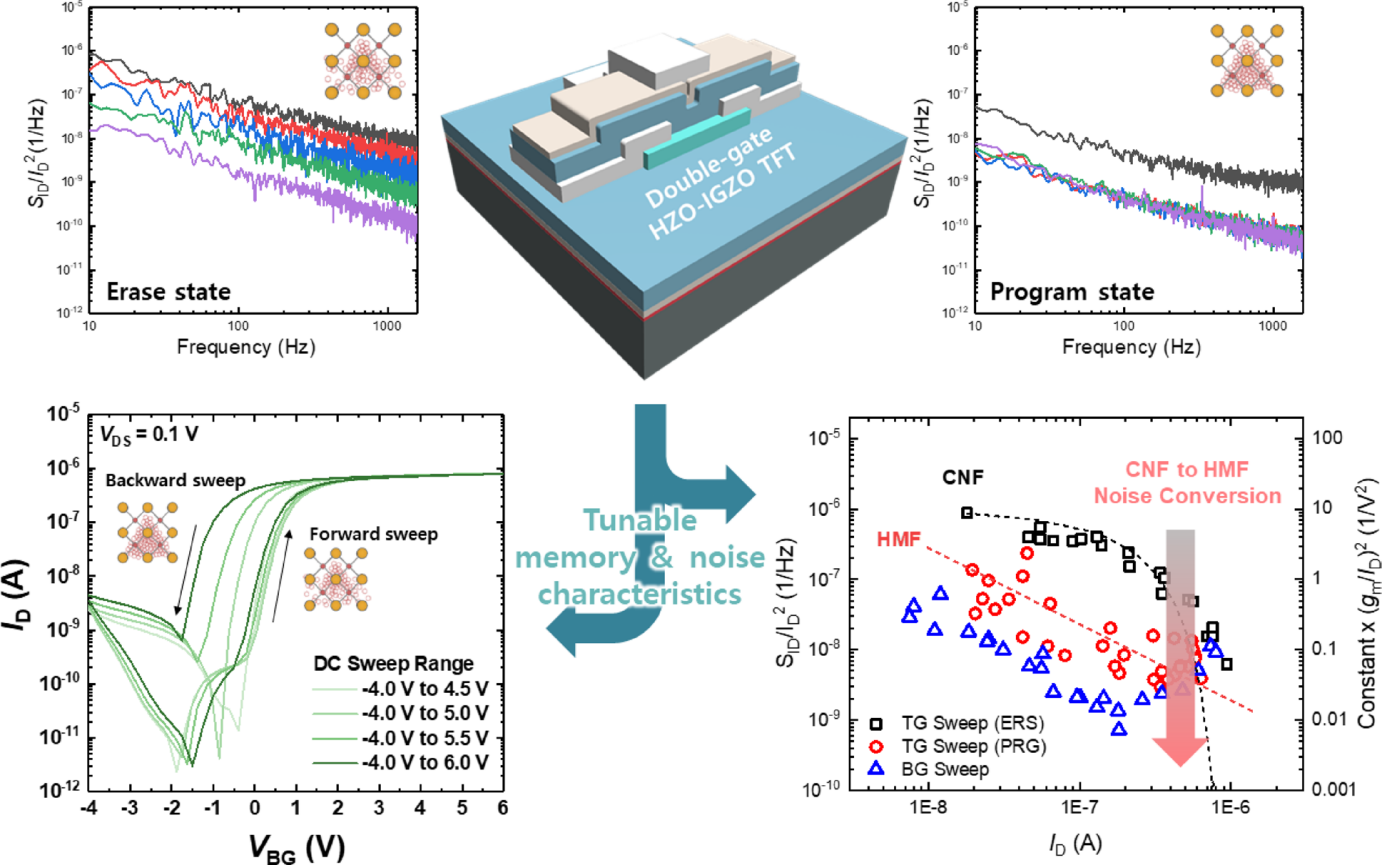

**Supplementary Information:**

The online version contains supplementary material available at 10.1186/s40580-025-00512-2.

## Introduction

High-k dielectrics have emerged as critical materials for next-generation semiconductor devices due to their high permittivity, CMOS compatibility, and versatile functionalities [1–4]. Initially introduced in advanced transistor gate stacks to replace SiO_2_ and scale down effective oxide thickness, high-k dielectrics like HfO_2_ have since found extensive applications beyond logic devices [5–10]. In resistive random-access memory (RRAM), HfO_2,_ Al_2_O_3_, and ZrO_2_ facilitate resistive switching through voltage-induced oxygen vacancy dynamics. Moreover, the landmark discovery of ferroelectricity in doped HfO_2_ thin films further diversified its role, paving the way for advanced memory devices such as ferroelectric RAM, ferroelectric tunnel junctions, and ferroelectric transistors. This rich interplay between high-k dielectric properties, defect-mediated switching, and ferroelectric polarization underscores the growing prominence in future logic-memory architectures and emerging computing paradigms.

Simultaneously, monolithic three-dimensional (M3D) integration has gained significant attention as it enables continued device scaling beyond conventional planar architectures [11, 12]. A crucial requirement for successful M3D integration is low-temperature processing to avoid degrading underlying device layers. Sputter-deposited high-k dielectric materials are particularly advantageous in this context because they enable wafer-scale deposition at low temperatures, ensuring compatibility with stringent thermal budgets of M3D integration. Additionally, oxide semiconductor thin-film transistors (TFTs), especially indium-gallium-zinc oxide (IGZO), meet these requirements due to their inherent compatibility with processes around 400 °C or lower [13, 14]. IGZO’s low thermal budget, excellent off-state resistivity, and stable carrier mobility make it an ideal semiconductor for integration above existing silicon circuits [15–20]. Importantly, combining IGZO channels with sputter-deposited HfO_2_-based gate dielectrics, including ferroelectric hafnium zirconium oxide (HZO) or ionic HfO_2_, allows the creation of fully back-end-of-line (BEOL)-compatible ferroelectric and ionic transistors. Such devices exhibit non-volatile memory characteristics and analog synaptic behavior, positioning them as promising candidates for neuromorphic computing applications.

Despite their significant potential, understanding the precise operation and switching mechanisms of HfO_2_-IGZO TFTs remains challenging due to intertwined phenomena involving ionic movement, charge trapping, and ferroelectric switching. Disentangling these complex processes necessitates characterization techniques sensitive to microscopic dynamics. Low-frequency noise (LFN) analysis, characterized by fluctuations in carrier number or mobility caused by charge trapping and defect interactions, emerges as a powerful method for probing these intricate mechanisms [21, 22]. A high sensitivity to subtle changes in ionic motion, trap dynamics, and ferroelectric polarization offers a pathway to clearly differentiate and quantify each underlying switching behavior.

In this work, we leverage LFN spectroscopy to systematically investigate the switching mechanism in a fully BEOL-compatible double-gate HfO_2_–IGZO TFT, alongside comprehensive material analysis. The device under study features HfO_2_ gate dielectrics and an IGZO semiconductor channel in a double-gate configuration, exemplifying an ionic TFT structure for BEOL memory applications. By correlating electrical switching behavior with noise spectra and material state observations, we aim to clarify the physical principles governing the device’s operation. The following sections present our findings, shedding light on whether the switching is primarily driven by ionic motion, charge trapping, ferroelectric polarization, or a synergistic interplay of these effects. Ultimately, gaining this insight is crucial for designing and optimizing HfO_2_-based oxide semiconductor transistors for reliable memory and neuromorphic computing at the back end of the line.

## Results and discussion

### Fabrication of double-gate TFT

**Fig. 1:**
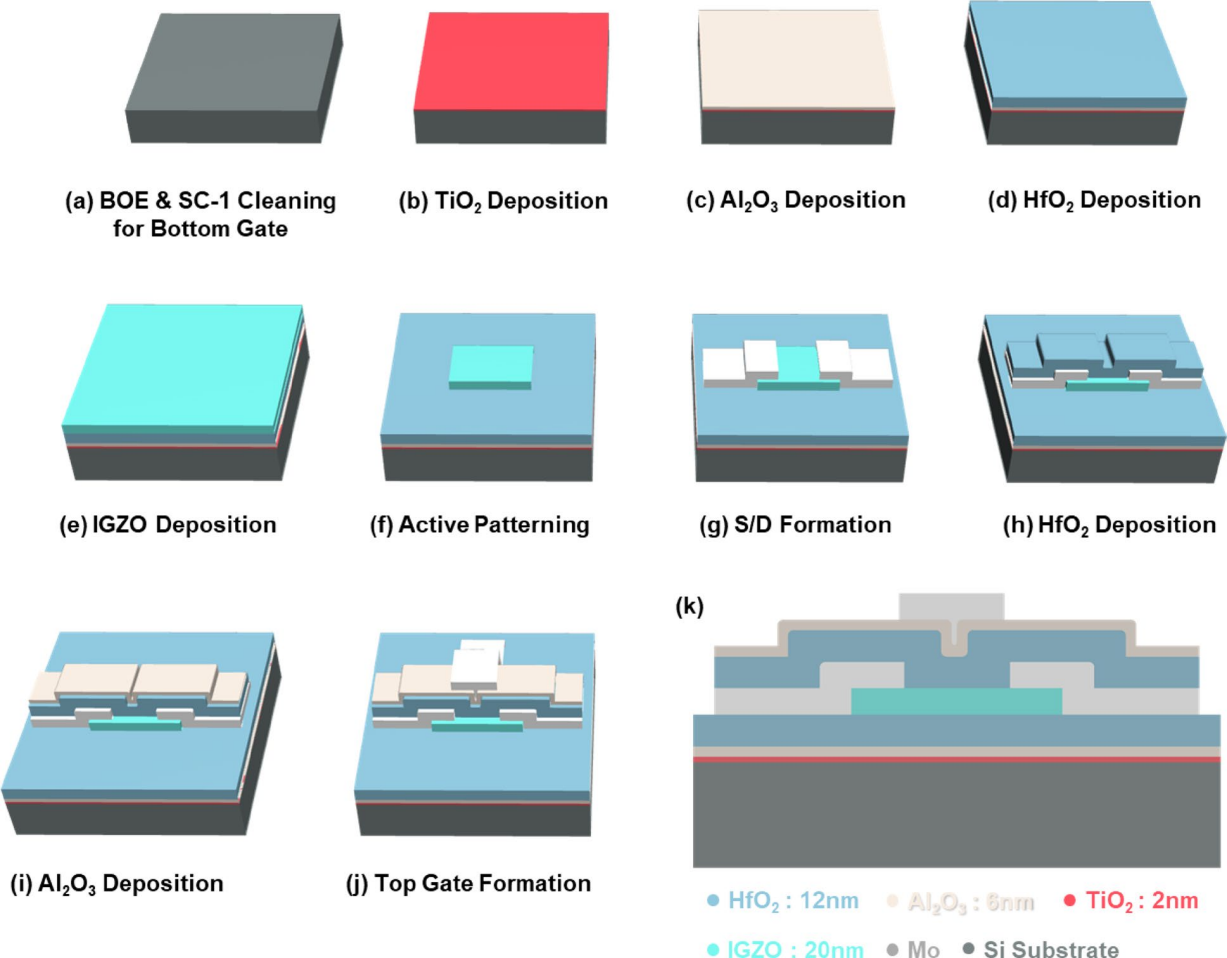
Fabrication process flow of the double-gate TFT. **a** Preparation of a highly doped *p*^+^ silicon substrate, serving as the bottom gate electrode. **b** Deposition of an ultrathin TiO_2_ (2 nm) interlayer to modulate oxygen vacancies at the bottom dielectric interface. Deposition of the bottom dielectric stack consisting of (**c**) Al_2_O_3_ (6 nm) and **d** HfO_2_ (12 nm) layers. **e** Formation of the IGZO active channel (20 nm) via RF sputtering. **f** Patterning of the IGZO layer to define the active channel region using selective wet-etching. **g** Deposition and patterning of molybdenum (Mo, ~ 33 nm) as source/drain electrodes. Deposition of the top dielectric stack, comprising (**h**) HfO_2_ (12 nm) and **i** Al_2_O_3_ (6 nm), matching the bottom dielectric structure. **j** Deposition and patterning of the top gate electrode (Mo, ~ 50 nm) through DC sputtering followed by a lift-off process. **k** Formation of contact holes and pads to finalize device structure, completing a fully symmetric dual-gate TFT structure suitable for ionic switching characterization

Double-gate TFTs were fabricated on a highly doped *p*^+^ silicon substrate, serving as the bottom gate electrode, following the process illustrated in Fig. 1. Initially, the substrate underwent standard cleaning (SC-1) and wet-etching with buffered oxide etchant (BOE) to remove any native oxide. Subsequently, gate dielectric layers composed of 2 nm TiO_2_, 6 nm Al_2_O_3_, and 12 nm HfO_2_ were sequentially deposited via radio frequency (RF) sputtering. An amorphous indium-gallium-zinc oxide (α-IGZO) layer was then deposited by RF sputtering with an Ar:O_2_ gas ratio of 9:1 using an IGZO 1:1:1:1 target, followed by annealing at 350 ℃ for 1 h under atmospheric conditions to enhance its electrical properties. The active channel region was patterned using selective wet-etching with diluted HCl, removing α-IGZO selectively. Next, molybdenum (Mo) source and drain electrodes (33 nm) were deposited by DC sputtering and patterned through a wet-etching process with an SC-1 solution (NH_4_OH:H_2_O_2_:DI = 1:8:64). Subsequently, top dielectric layers of HfO_2_ (12 nm) and Al_2_O_3_ (6 nm) were deposited by RF sputtering, matching the thickness of the bottom dielectric stack. Finally, the top gate electrode (50 nm Mo) was formed using DC sputtering followed by a lift-off process, and contact holes and pads were fabricated as the concluding step. Notably, the entire fabrication process was conducted at temperatures below 350 ℃, satisfying the thermal budget constraints necessary for M3D integration.

### Structure and material characteristics of double-gate TFT

**Fig. 2:**
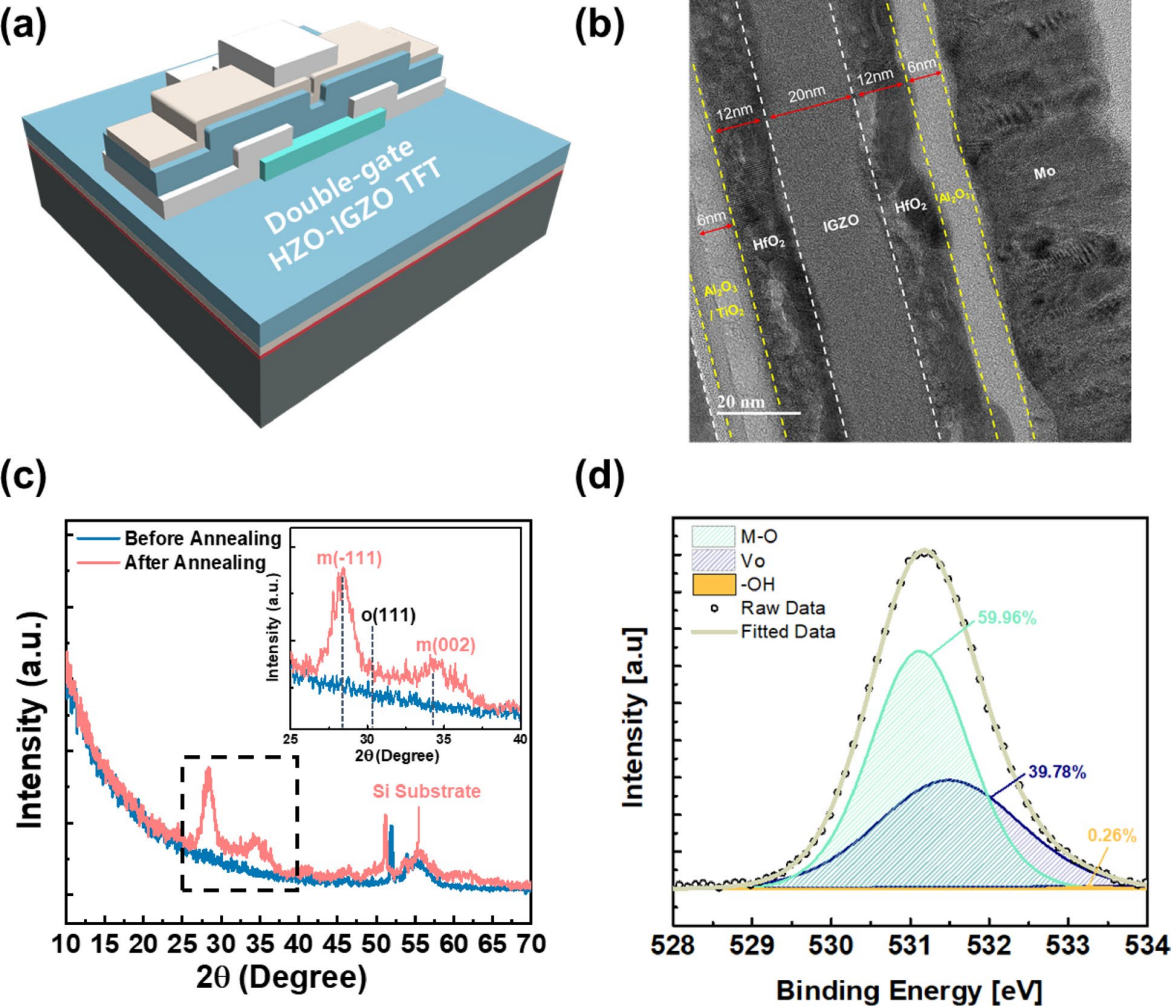
Structural and material characterization of the double-gate TFT. **a** Three-dimensional schematic illustration of the final device structure, highlighting the uniformly deposited high-k dielectric stacks and the presence of the ultrathin TiO_2_ interlayer at the bottom interface. **b** Cross-sectional TEM image clearly resolving each dielectric layer and their sharp interfaces, confirming successful low-temperature processing. **c** XRD spectra of the HfO_2_ gate dielectric without distinct ferroelectric crystalline phases. The inset magnifies the 25°–40° range, revealing no diffraction peaks related to the o-phase. **d** XPS O1s spectra of IGZO, deconvoluted into three components: lattice oxygen, oxygen vacancies, and surface hydroxyl or adsorbed oxygen species

Figure 2a shows the 3D schematic of the double-gate transistors. The device is a double-gate TFT with a multilayer gate dielectric stack on both the bottom and top gates. In the schematic (panel a), the bottom gate electrode (on the substrate) is insulated from the semiconductor by a composite high-κ dielectric stack consisting of TiO_2_ (2 nm), Al_2_O_3_ (6 nm), and HfO_2_ (12 nm). This bottom gate stack is engineered to achieve a high overall dielectric constant and good interface quality. Above the bottom dielectric lies the active channel: a 20 nm IGZO layer. For IGZO channels, a thinner film generally leads to a more positive threshold voltage (*V*_th_) and improved electrical characteristics such as subthreshold swing (SS) and on-current (*I*_ON_). However, considering the electrostatic coupling between the top and bottom gates inherent in the double-gate TFT structure, the channel thickness was optimized to 20 nm to ensure independent gate control while maintaining good device performance [23, 24]. To evaluate the electrical performance of the IGZO channel, transfer and output characteristics were confirmed using a single bottom gate TFT structure, and the corresponding results with schematic are presented in Fig. S1. On top of the IGZO, the device includes a symmetric top gate dielectric composed of HfO_2_ (12 nm) and Al_2_O_3_ (6 nm), which mirrors the thicker two layers of the bottom stack. Finally, a top gate metal (Mo) is stacked over the top dielectric. Thus, the IGZO channel is sandwiched between two gate stacks, each incorporating HfO_2_ and Al_2_O_3_ layers. The TiO_2_ is used only at the bottom interface as an ultrathin interfacial layer, whereas the top gate stack omits this TiO_2_ layer. In our device design, TiO_2_ was intentionally incorporated into the bottom gate stack based on its high oxygen bond dissociation energy, which enables spontaneous oxidation at the electrode interface and the natural formation of a thin interfacial layer [25–27]. Given that annealing process is essential after the formation of the bottom gate dielectric stack and the IGZO channel to enhance channel mobility, the insertion of TiO_2_ as a bottom gate dielectric allows us to effectively observe the role of TiO_2_ in increasing oxygen vacancy concentration while simultaneously enhancing device performance. This dual-gate configuration enables electrostatic control of the channel from both sides, allowing for the investigation of the effects of different gate stacks on the electrical characteristics within a single device.

Figure 2b shows a cross-sectional transmission electron microscopy (TEM) image of the fabricated TFT, confirming the stratified structure of the deposited layers (Energy Dispersive Spectroscopy (EDS) data is shown in Fig. S2). The TEM cross-section reveals well-defined, sharp interfaces between each successive layer. This suggests that the low-temperature deposition and processing steps successfully preserved the intended layer boundaries. Figure 2c shows X-ray diffraction (XRD) data collected from the HfO_2_ layer of the device’s gate stack. The XRD pattern is characteristic of an amorphous film: it shows only a broad, low-intensity hump instead of sharp diffraction peaks. No distinct peaks corresponding to crystalline HfO_2_ phases are observed before the annealing; in particular, even after the annealing the pattern lacks any reflections that would indicate the presence of the ferroelectric orthorhombic (o-) phase of HfO_2_. Note that o-phase, especially o(111), HfO_2_ typically yields a diffraction peak around 2θ ≈ 30.5°, attributed to the non-centrosymmetric Pca2_1_ structure [28]. The diffraction peaks observed at approximately 28.4° and 34.5° after annealing are attributed to the (-111) and (002) planes of monoclinic-phase (m-phase) HfO_2_, respectively. These results suggest that the HfO_2_ film attains a thermodynamically stable poly-crystalline phase upon thermal annealing [29, 30]. In doped HfO_2_ systems (such as HfO_2_ doped with Al, Zr, Si, etc.), the emergence of ferroelectric behavior is correlated with the formation of a polar orthorhombic phase, often achieved through appropriate doping and annealing treatments [31]. However, in the HfO_2_ film used in this study, no such peak is observed, indicating that it should behave as a normal high-k dielectric without spontaneous polarization. This is due to the low temperature of the annealing process.

Additionally, we investigate the IGZO film characteristics. Figure 2d shows the X-ray photoelectron spectroscopy (XPS) spectrum of the IGZO film, focusing on the O1s core-level region. The high-resolution O1s spectrum is deconvoluted into three main components, reflecting different chemical environments of oxygen in the film. The first component, centered at a binding energy ~ 529–530 eV, corresponds to fully boned O atoms in M–O–M lattice (In, Ga, Zn metal oxide bonds). A second O1s feature appears at a slightly higher binding energy (~ 531 eV), which is attributed to oxygen in oxygen-deficient environments, i.e. oxygen atoms neighboring an oxygen vacancy or otherwise in a reduced state.^15^ The third component, at an even higher binding energy (~ 532 eV), is assigned to surface hydroxyl groups (OH^−^) or adsorbed molecular oxygen/water [32]. These OH species likely form from ambient moisture reacting with under-coordinated metal sites or oxygen vacancies at the film surface. The XPS analysis therefore confirms that the IGZO film contains appreciable oxygen vacancy content, alongside the expected M–O bonds, and some surface OH contamination. Such oxygen vacancies are commonly generated during post-deposition anneals in reducing conditions [33].

### Electrical characteristics of double-gate TFT

**Fig. 3:**
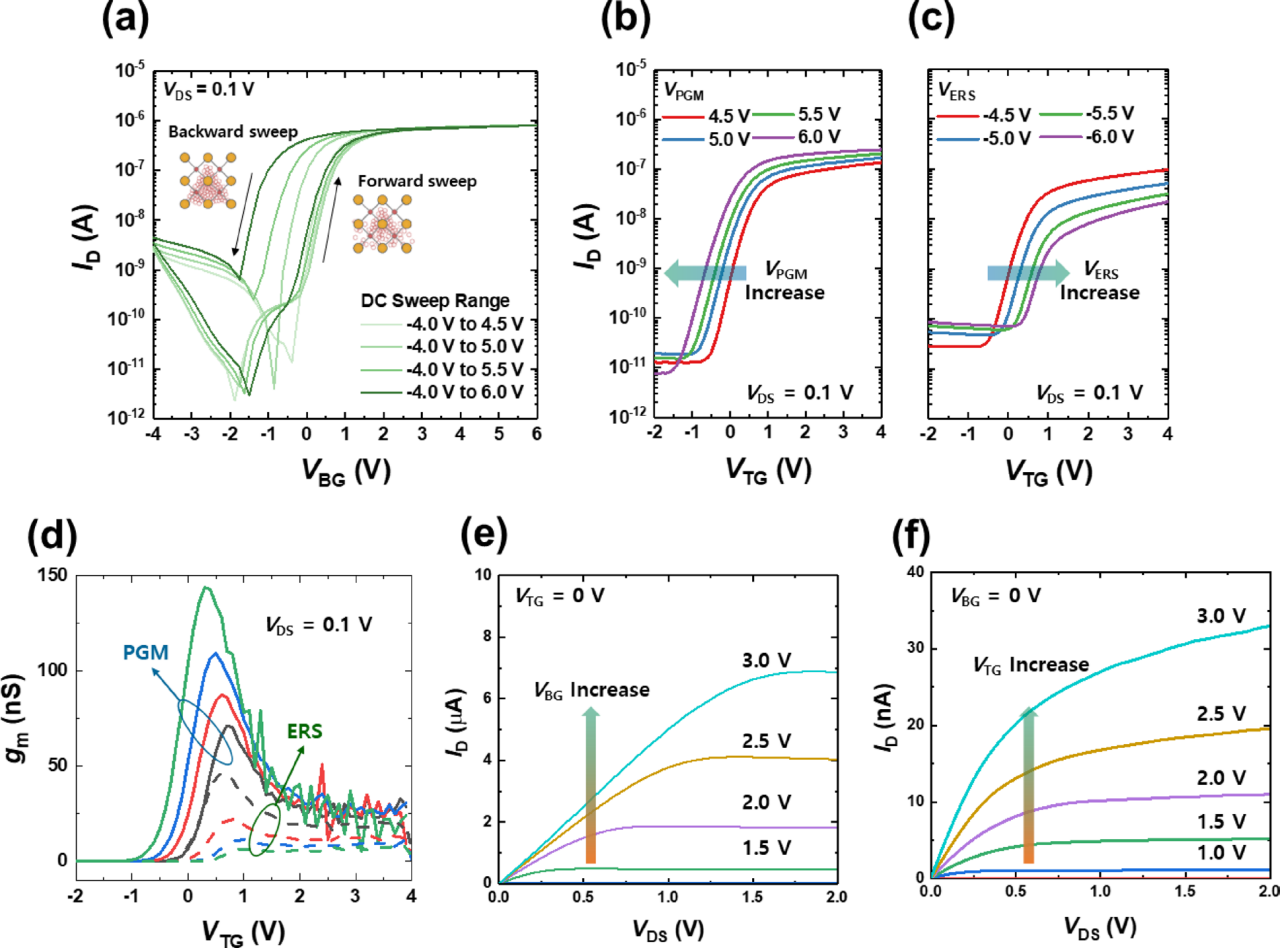
Electrical characterization of double-gate TFT. **a** Transfer characteristics (*I*_D_-*V*_BG_) exhibiting clear counterclockwise hysteresis, indicating ionic migration and trapping dynamics in the bottom dielectric. Insets depict ionic vacancy migration scenarios under positive and negative bias conditions. **b**, **c** Top-gate transfer curves (*I*_D_-*V*_TG_) following programming and erasing conditions, demonstrating significant modulation of *V*_th_, SS, and drive current by bottom-gate induced states. **d** Extracted transconductance (*g*_m_) clearly increased under programmed conditions due to enhanced bulk conduction. **e**, **f** Output characteristics (*I*_D_-*V*_DS_) showing the modulation of saturation current and channel conductance through bottom and top gate biases

Now, we investigate the electrical characteristics of the double-gate transistors. Figure 3a shows the transfer characteristics (*I*_D_-*V*_BG_) of the double-gate transistors by sweeping the bottom-gate voltage (*V*_BG_) while the top gate voltage (*V*_TG_) is grounded. These bottom-gate transfer characteristics exhibit a pronounced counterclockwise hysteresis loop. Such hysteresis is a strong indicator of field-induced ionic migration in the gate dielectric. As *V*_BG_ is swept positively (forward sweep), the electric field drives positively charged oxygen vacancies within the HfO_2_ dielectric toward the IGZO channel interface, effectively increasing electron concentration in the channel. On the return sweep (reducing *V*_BG_), these oxygen vacancies relax only slowly away from the interface. This behavior results the fact that some of the vacancies that migrated during the forward sweep become trapped in interface states near the channel, making it difficult for them to diffuse back into the bulk. Additionally, the electrons accumulated at the interface during the forward sweep can partially screen the applied electric field, further hindering the migration of oxygen vacancies [34, 35]. Threfore the channel remains accumulated for longer, yielding higher drain current than in the forward sweep at equivalent *V*_BG_. This delayed backward movement of ionic charge maintains electron accumulation during the reverse sweep, resulting in a counterclockwise hysteresis and an apparent memory effect in the transfer curve. In principle, although oxygen vacancy migration can occur across all underlying dielectric layers under an applied electric field, it is the HfO_2_ layer, directly interfacing with the IGZO channel, that plays a dominant role in modulating the channel carrier concentration. Therefore, we attribute the observed counterclockwise hysteresis behavior primarily to the migration of oxygen vacancies within the HfO_2_ dielectric. The observed hysteresis supports the ionic modulation mechanism: the *V*_th_ shifts are governed not just by electronic charge at the interface, but by the migration of oxygen ions/vacancies in the HfO_2_ gate insulator under an applied field.

Figure 3b, c show the top-gate transfer characteristics (*I*_D_–* V*_TG_) measured after applying program and erase biasing of the bottom gate, respectively. Note that no hysteresis is observed in the double sweep measurement (Fig. S3). In these measurements, the device was first subjected to an out-of-plane gate pulse on *V*_BG_ to alter the state of the channel, and then the top-gate transfer was swept. The results show that the prior bottom-gate biasing dramatically reconfigures the transistor behavior: the *V*_th_, SS, and transconductance of the top-gate sweep are all significantly modulated between the programmed and erased states. In the programmed state (Fig. 3b), which follows the application of a positive *V*_BG_ pulse, the top-gate transfer curve shifts to lower *V*_th_ and higher conductivity. Additionally, *I*_ON_ is markedly increased, and the subthreshold slope becomes steeper. Physically, a positive back-gate pulse accumulates extra electrons in the IGZO channel region via the migration of oxygen vacancies toward the channel. In effect, the bottom gate pulse programs the channel into a more conductive state by enriching it with oxygen vacancies, which act as electron donors, or by filling trap states. This defect-induced field effect by the back gate is strongly coupled to the channel: the bottom gate serves as an electron supplier, raising the channel’s electron concentration and conductivity. Consequently, when the top gate is swept afterward, it controls a channel that is already pre-conditioned to conduct. This is evidenced by the increased drive current and the enhanced transconductance in the programmed transfer curve, which will be discussed further below. In the erased state (Fig. 3c), produced by applying a negative *V*_BG_ pulse, the opposite behavior is observed. The top-gate transfer curve shifts toward higher *V*_th_ and reduced channel conductivity. Here, a negative back-gate bias has pulled oxygen vacancies away from the channel interface or pushed the channel into depletion, effectively reducing the free carrier concentration in the IGZO. The memory-like behavior demonstrated by these sweeps—i.e., the retention of a programmed or erased state in the channel that persists during the subsequent top-gate operation—underscores the strong electrostatic coupling between the two gates. Additionally, Supplementary Fig. S4b presents the retention characteristics corresponding to the memory-like behavior of the device, illustrating the stability of the programmed and erased states over time. This result indicates that the distribution of oxygen vacancies and charge rearrangement within the device remain stable, supporting the feasibility of non-volatile memory applications. The fully programmed and erased conditions used for retention measurements were determined based on switching speed measurements conducted on the bottom gate TFT, as shown in Fig. S4(a). These speed measurements indicate that the observed hysteresis behavior originates from ionic migration, which inherently requires relatively slow response times and high operating voltages. Figure 3d plots the transconductance (*g*_m_) extracted from the top-gate transfer curves under the various back-gate bias conditions. The programmed condition yields a markedly higher *g*_m_ and *I*_ON_ than the erased or initial conditions. This enhancement in g_m_ implies that the IGZO channel contains a higher electron density such that the top gate can modulate the channel more efficiently after programming. Figure 3e, f show the output characteristics (*I*_D_–* V*_DS_) of the transistor measured at different *V*_BG_s and *V*_TG_s values, respectively. These output curves illustrate how the drive current and saturation behavior of the TFT can be electrostatically tuned by the bottom gate and top gate.

### Oxygen vacancy characterization

**Fig. 4:**
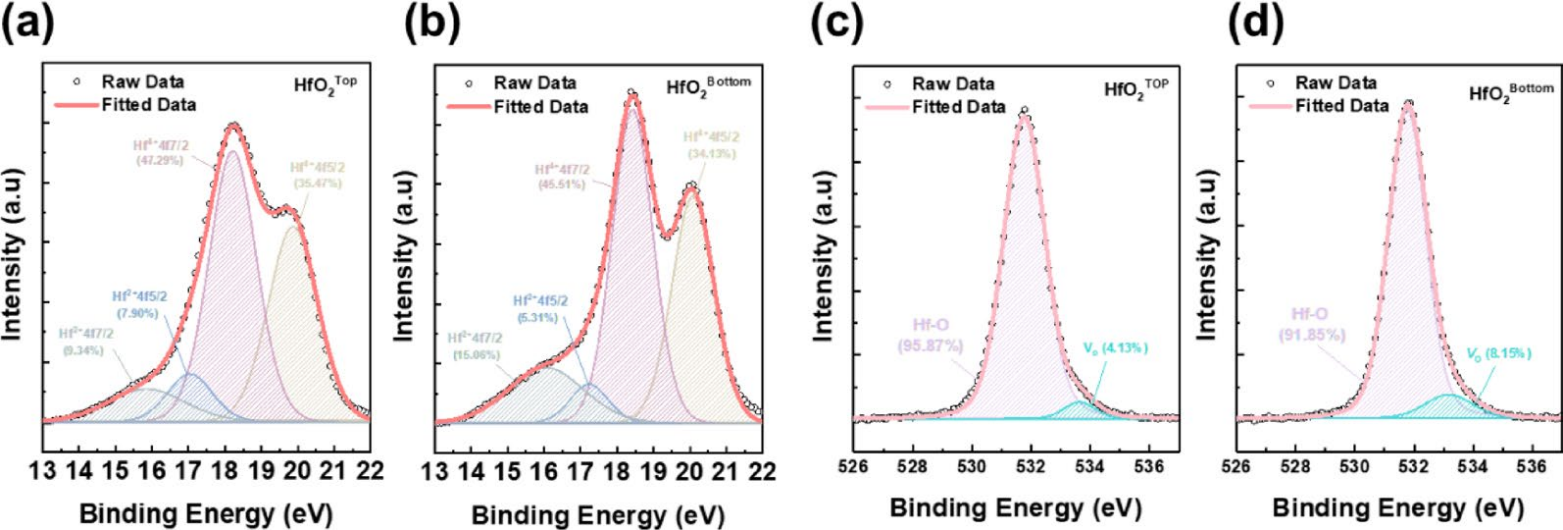
XPS characterization of oxygen vacancy distributions. High-resolution XPS spectra comparing the Hf4f (**a**, **b**) and O1s (**c**, **d**) core-level regions for the top-gate and bottom-gate dielectric stacks. Clear evidence of increased oxygen vacancies at the bottom-gate stack due to TiO_2_-induced oxygen scavenging is observed through prominent vacancy-related peaks

Now, we investigate the origin of different gate responses at the top and bottom gate voltage sweep. Figure 4a, b shows XPS spectra of Hf4f core levels for the top-gate and bottom-gate regions of the investigated HfO_2_ dielectric layer, respectively, elucidating distinct distributions of oxygen vacancies within the oxide layers. The measured spectra exhibit two characteristic sets of doublet peaks, each corresponding to the spin–orbit coupling components Hf 4f7/2 and Hf 4f5/2 [36]. Specifically, the peaks centered around approximately 16.5–17.0 eV and 18.0–18.5 eV are typical signatures of stoichiometric HfO_2_, representing Hf fully coordinated with oxygen atoms (Hf–O bonds). On the other hand, the lower binding energy peaks, observed approximately between 15.5–16.0 eV and 17.5–18.0 eV, correspond to Hf 4f states associated with oxygen-deficient bonding environments, indicative of the presence of oxygen vacancies in HfO_2_ lattices. From quantitative analysis of spectra, the proportion of Hf states bonded to oxygen vacancies in the top gate accounts for approximately 17.2%, whereas the proportion increases notably to approximately 20.4% in the bottom gate. This substantial increment in vacancy-related states clearly indicates an elevated concentration of oxygen vacancies near the bottom-gate interface region, directly associated with the TiO_2_/Al_2_O_3_/HfO_2_ stack structure. The larger portion of the oxygen vacancy in the HfO_2_ at the bottom gate is further demonstrated by O1s spectra analysis in Fig. 4c, d. The distributions of the O1s and Hf4f spectra according to the position across the overall device structure are presented in Fig. S5 and Fig. S6, respectively. These oxygen vacancies are not just static defects; in oxide dielectrics they can behave as mobile ionic charge carriers under an electric field [37, 38]. In the fabricated HfO_2_–IGZO TFT, when a gate bias is applied, these vacancies, which can be thought of as positively charged defects when ionized, are able to migrate within the HfO_2_ layer. This phenomenon is a form of ionic transport in the gate dielectric. This bias-driven movement of oxygen vacancies is analogous to that in ionic memory devices [39] and has been widely recognized as a cause of hysteresis in oxide TFTs. Understanding this mechanism is crucial for interpreting the device’s behavior and aligns with other IGZO–HfO_2_ TFT studies that report hysteresis stemming from ionic defect movement rather than ferroelectric switching [40, 41]. Furthermore, the evident difference in oxygen vacancy concentration between top-gate and bottom-gate HfO_2_ can be attributed to the TiO_2_ layer's intrinsic property as an oxygen scavenger [42, 43]. TiO_2_, known for its affinity to form sub-stoichiometric phases (TiO_2-x_), actively attracts and captures oxygen from neighboring oxide layers. Such a chemical interaction leads to a depletion of oxygen from the adjacent HfO_2_ layer, resulting in an increased density of oxygen vacancies at the HfO_2_ interface near TiO_2_. Consequently, this spatial distribution of oxygen vacancies critically influences the electrical characteristics of the fabricated device. The hysteresis mechanism induced by TiO_2_-related ionic migration can also be supported by the Positive-Up-Negative-Down (PUND) measurement results shown in Fig. S7. As confirmed by the XRD analysis in Fig. 2c, the HfO_2_ used in the device acts as a purely dielectric layer. In the absence of TiO_2_, the HfO_2_ and Al_2_O_3_ stacks exhibit no switching current in the PUND measurements, indicating the absence of ferroelectricity. In contrast, when TiO_2_ is included, a weak ferroelectric-like behavior emerges, which can be attributed to ionic defect movement facilitated by an increased concentration of oxygen vacancies [44–46].

**Fig. 5:**
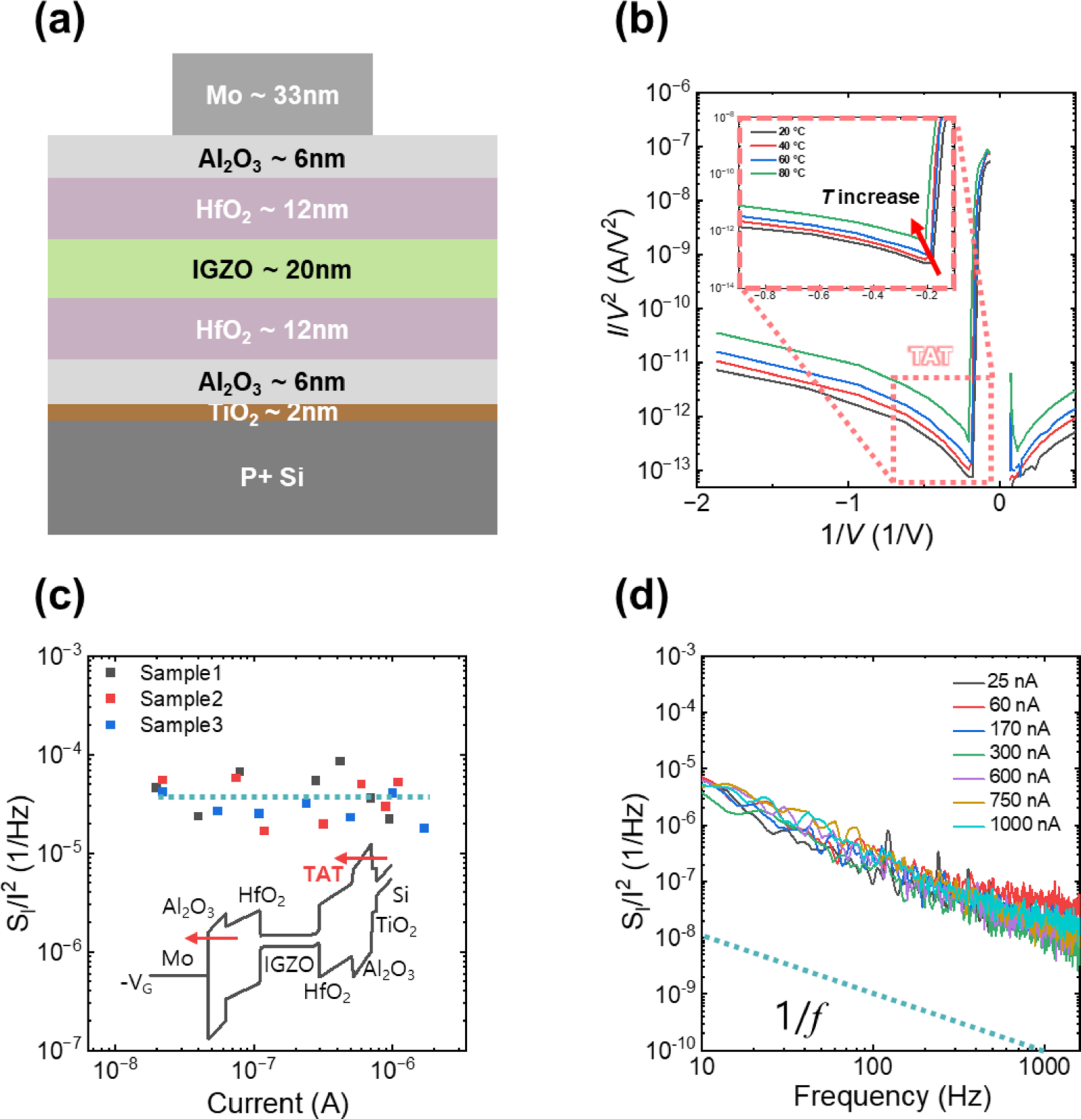
**a** Cross-sectional schematic of the fabricated capacitor structure. **b** Plot of *I*/*V*^2^ versus 1/*V* at various temperatures, demonstrating exponential dependence characteristic of TAT conduction through defect states in the dielectric. **c** *S*_I_/*I*^2^ versus current, consistently displaying 1/*f* noise behavior attributed to trapping-detrapping events. The inset shows energy band diagram of fabricated capacitor stacks at negative voltage. **d** *S*_I_/*I*^2^ versus frequency at varying current. The fitting line demonstrates that the 1/*f* noise behavior of the capacitor can be explained by TAT

To further elucidate the oxygen vacancy dynamics and their contribution to electrical transport, we fabricated capacitor structures with identical dielectric stacks as the TFT (Fig. 5a) and conducted LFN analysis. This approach isolates the intrinsic dielectric characteristics, specifically probing the defect-mediated tunneling mechanism within the HfO_2_ layer. Figure 5b shows the *I*/*V*^2^ versus 1/*V* plot of the capacitor measured under varying bias and temperature conditions. The obtained curves exhibit a clear exponential dependence, characteristic of trap-assisted tunneling (TAT) conduction, suggesting that electrons tunnel through localized defect states created by oxygen vacancies within the dielectric [47]. Figure 5c shows the frequency dependence of normalized current noise spectral density (*S*_I_/*I*^2^) at different bias conditions to extract current values ranging from 10^–8^ to 10^–6^ A. All measured conditions reveal typical 1/*f* noise characteristics, consistent with trapping-detrapping events within the dielectric layer (Fig. 5d). Specifically, under TAT conduction conditions, oxygen vacancies act as localized traps facilitating tunneling [48, 49]. Such trapping events give rise to fluctuations in tunneling probability and thus contribute to 1/*f* noise spectra associated with charge trapping/detrapping processes [50]. These results collectively provide comprehensive evidence that oxygen vacancy-induced traps are primarily responsible for the TAT conduction and its associated 1/*f* noise behavior in the high-k dielectric structures.

### LFN characteristics of double-gate TFTs

**Fig. 6:**
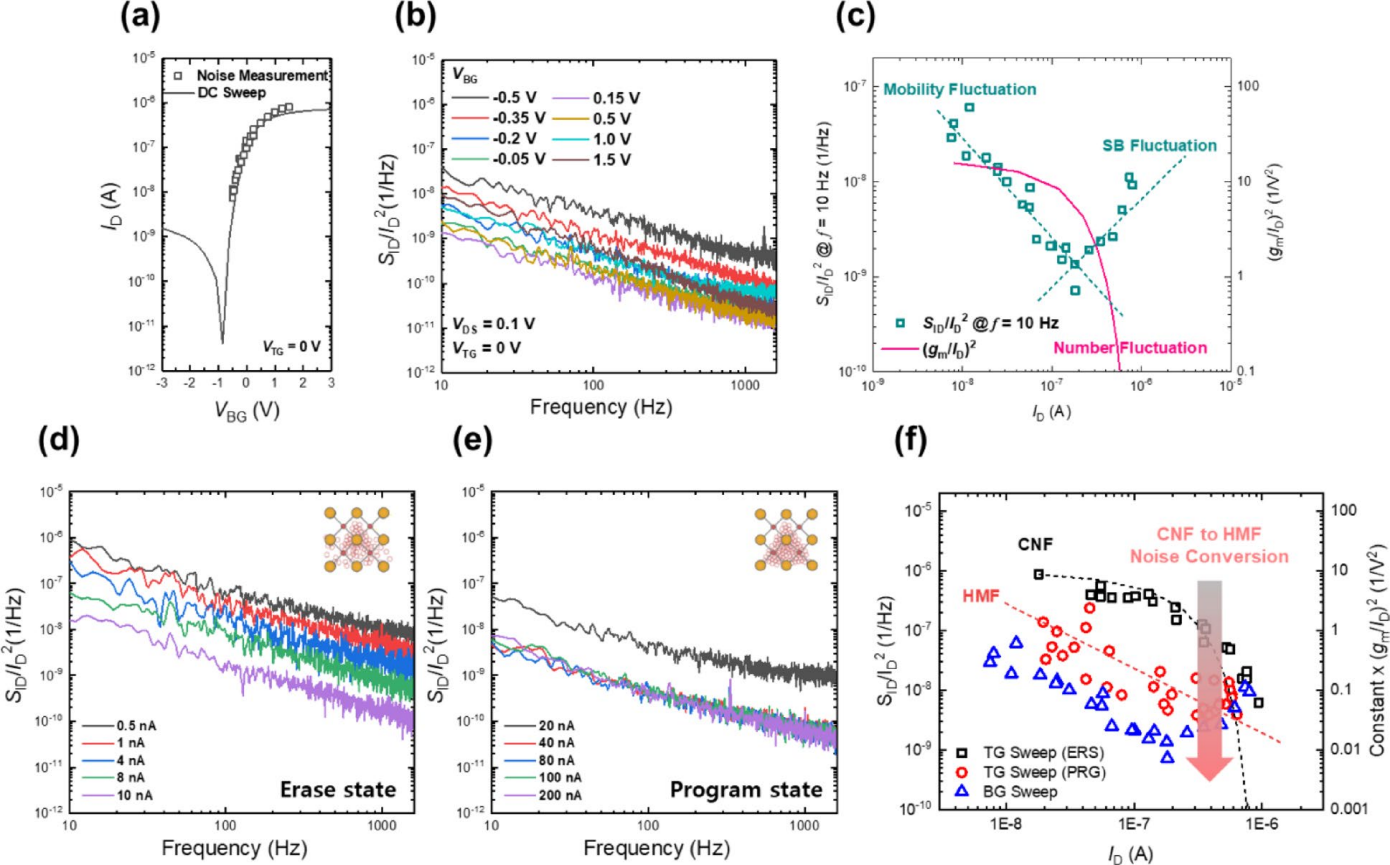
**a** Comparison between noise spectra and corresponding DC *I*-*V* characteristics demonstrating measurement consistency. **b** *S*_ID_/*I*_D_^2^ versus frequency at varying bottom-gate biases. **c** *S*_ID_/*I*_D_^2^ sampled at 10 Hz versus *I*_D_, showing transition from HMF to Schottky barrier fluctuation noise with increased *V*_BG_. *S*_ID_/*I*_D_^2^ versus frequency measured at programmed (**d**) and erased (**e**) states, by changing the *V*_TG_. **f** *S*_ID_/*I*_D_^2^ sampled at 10 Hz versus *I*_D_, showing transition from CNF to HMF noise conversion driven by oxygen vacancy modulation in the dielectric stack

Based on above analysis, we investigate the LFN characteristics of double-gate TFTs. First, the LFN of the transistors is measured by changing the *V*_BG_. The measurement setup for the LFN spectroscopy is described in Method section. As shown in Fig. 6a, the noise measurements are well-matched with the corresponding DC *I*-*V* data, confirming the stability and reliability of the LFN measurement process. This match assures that the observed LFN does not stem from experimental artifacts or drift but is a reliable reflection of the intrinsic noise characteristics of the device under various operating conditions. Figure 6b shows the evolution of the noise power spectral densities (PSDs) versus frequency at various *V*_BG_s. For all bias conditions, the noise exhibits clear 1/*f* noise characteristics. The observed 1/*f* noise behavior indicates that the primary noise source in the device arises from fluctuations in charge carrier number and mobility, which are typically associated with trap states and defects in the channel or gate dielectric [51, 52]. To identify the specific noise sources, the carrier number fluctuation (CNF) and Hooge’s mobility fluctuation (HMF) models are compared with the experimental results (Fig. 6c). The CNF model is given as [53]

$$\frac{{S}_{ID}}{{{I}_{D}}^{2}}={(\frac{{g}_{m}}{{I}_{D}})}^{2}{S}_{Vfb}$$

where *S*_ID_/*I*_D_.^2^ is the drain current normalized PSD, *S*_Vfb_​ represents the PSD associated with fluctuations in the flat-band voltage due to the CNF, which can be defined as [53]

$${S}_{Vfb}=\frac{{q}^{2}{N}_{t}kT\lambda }{WL{{C}_{Ox}}^{2}f}$$

*N*_t_ represents the volume trap density, $\lambda$ is the oxide tunneling attenuation distance, and *C*_OX_ is the gate oxide capacitance per unit area. On the other hand, the HMF model is given as [54]

$$\frac{{S}_{ID}}{{{I}_{D}}^{2}}=\frac{{\alpha }_{H}{\mu }_{eff}2kT}{f{L}^{2}{I}_{D}}$$

where $\alpha$_H_ represents Hooge’s parameter and ${\mu }_{eff}$ is the effective carrier mobility. At lower *V*_BG_, the noise is dominated by the HMF model, which is associated with variations in the carrier mobility due to the scattering at the bulk of the channel, often due to ionization and trapping at defects or oxygen vacancies. As the current increases with higher *V*_BG_, there is a transition toward contact noise is observed, where the primary noise source becomes related to the Schottky barrier fluctuation at the source/drain electrodes.

Figure 6d and e show the drain current normalized PSD versus frequency of the transistor measured at a fixed bottom gate bias while varying the top gate bias. During the measurement, the bottom gate was grounded (*V*_BG_ = 0 V) and a drain voltage of 0.1 V was applied. The gate voltage was applied to the top gate, corresponding to the target drain current. The device was characterized in two distinct states—programmed and erased—achieved through voltage pulses applied to the bottom gate. Although both states exhibit the characteristic 1/*f* noise behavior, the PSD dependence on the *V*_BG_ differs significantly between them. In the erased condition, the noise spectra adhere to the CNF model, indicating that the dominant noise source is carrier trapping and detrapping events involving defects located at the top-gate oxide/IGZO interface. This behavior implies that, under erased conditions, the IGZO channel is depleted of carriers. Consequently, conduction primarily occurs near the interface between the IGZO channel and the top-gate dielectric, where the limited number of available carriers makes the channel more susceptible to trapping events. As a result, fluctuations in the number of carriers strongly influence conduction, leading to higher noise levels dominated by CNF. The trap density estimated from the CNF model in this case is presented in Fig. S8. To evaluate the trap density, the dielectric constant of the top stacks was extracted as 11.81 through measurement and calculation, and assuming the dielectric constant of SiO_2_ to be 3.9, the equivalent oxide thickness (EOT) was calculated to be approximately 5.94 nm. (For the bottom layers excluding TiO_2_, the corresponding values were 12.71 and 5.52 nm, respectively, showing a difference compared with the top stacks.)

In contrast, the programmed state displays a clear shift in noise characteristics, conforming instead to the HMF model. This shift suggests that the programming process induces a substantial increase in mobile carriers within the IGZO channel through the migration and charging of oxygen vacancies in the bottom gate dielectric (HfO_2_). The higher carrier density not only enhances conductivity but also moves the conductive channel away from the top-gate dielectric interface toward the bulk of the IGZO channel. Consequently, the channel electrons predominantly conduct through bulk pathways, significantly reducing their direct interaction with interface trap states. Due to this spatial redistribution of carriers into bulk regions, the overall impact of individual carrier trapping and detrapping events at the top-gate interface is markedly diminished. Instead, random fluctuations in carrier mobility—caused primarily by scattering centers, including charged defects related to oxygen vacancies within the bulk dielectric—emerge as the dominant source of noise. Therefore, in the programmed state, noise levels are generally reduced, and the underlying physical mechanism transitions from CNF to bulk mobility fluctuation (Fig. 6f). For the evaluation of the HMF model, the effective mobility calculated from the measurement results was 2.4 cm^2^V^−1^s^−1^, and the corresponding estimated constant was approximately 3.08 × 10^–3^.

The findings of this study significantly deepen our understanding of ionic switching dynamics driven by oxygen vacancy modulation within high-k dielectric materials. By precisely identifying how oxygen vacancy concentration and migration influence electrical and noise characteristics, this research provides a crucial framework for optimizing device performance. The demonstrated control over ionic dynamics through dielectric engineering paves the way for developing more reliable and scalable memory devices, particularly for neuromorphic computing and M3D integration, where precise ionic manipulation can enable innovative functionalities.

## Conclusion

In this study, we investigated a double-gate IGZO transistor with distinct oxygen vacancy concentrations in the HfO_2_ gate dielectrics of the top and bottom gates. Material analyses confirmed differing oxygen vacancy levels between top and bottom gate oxides due to the oxygen scavenging role of TiO_2_ at the bottom gate oxide stacks, enabling memory operation via bottom gate modulation. Electrical characterization demonstrated memory window operation, while low-frequency noise analysis clarified the underlying carrier dynamics. In the erased state, noise spectra aligned with the CNF model, dominated by interface trap-related carrier trapping near the top-gate oxide interface. Conversely, the programmed state exhibited a shift to the HMF model due to increased bulk carrier conduction induced by oxygen vacancies in the bottom dielectric, significantly reducing interface trapping effects. This asymmetry between the top and bottom gates not only allowed control over the memory window but also provided a means to tune critical device parameters such as subthreshold swing and *g*ₘ. These findings underscore the potential of gate dielectric asymmetry for optimizing device performance and enabling advanced memory applications.

## Methods

### XRD analysis

Qualitative analysis was conducted to elucidate the operating mechanism of the device and the characteristics of the thin film. Firstly, phase distribution of HfO_2_ layer according to heat treatment was investigated through grain incidence X-ray diffraction (GIXRD) analysis. It was executed on the two samples, which had the same stack as the bottom TFT of the device, before and after annealing process. The diffraction patterns for each sample were obtained by X-ray powder diffraction X’Pert PRO MRD system (Phillips Corporation) with a Cu Kα (*λ* = 1.5406 Å) in the 2θ angle from 10º to 90º with a step size of 0.02º.

### XPS measurements

X-ray photoemission spectroscopy (XPS) was used to confirm the distribution of the oxygen deficiency concentration and its effects. The XPS spectra were recorded with a Thermo Fisher Scientific Nexsa G2 system. It was performed on the sample that had the same stack as the device and went through the same process. The spectrometer was referenced and calibrated using C1s main peak at 284.8 eV. We carried out the XPS fitting for the primary data with the software CasaXPS to estimate and subtract the background signal along the selected range of binding energy, resulting in using Shirley background.

### LFN measurement

To investigate the LFN characteristics of the double-gate IGZO TFT, we employed a precise measurement setup incorporating a semiconductor parameter analyzer (B1500A), a low-noise current amplifier (SR570), and a signal analyzer (35670A). The measurement methodology is designed to capture the drain current fluctuations with high sensitivity and accurately extract the power spectral density (PSD) through Fast Fourier Transform (FFT) analysis. In our setup, the gate bias is applied using the B1500A, ensuring precise control over the transistor’s operating point. Simultaneously, a constant drain bias of 0.1 V was applied via the SR570, which served both as a biasing source and as a low-noise current amplifier to enhance the signal-to-noise ratio during noise measurements. The amplified drain current signal is then fed into the 35670A signal analyzer, which processes the time-domain fluctuations and extracts the frequency-dependent noise characteristics using FFT-based PSD calculations. The selection of this measurement technique is based on the fundamental principle that, in FETs, the primary source of signal fluctuations originates from variations in the drain current [55–57]. Consequently, monitoring the drain current noise spectrum provides valuable insight into the underlying noise mechanisms, including carrier trapping/detrapping processes, Schottky barrier fluctuations, and bulk semiconductor noise contributions. In this study, the frequency range for LFN measurements was carefully chosen to cover the spectrum where 1/*f* noise is most pronounced. Specifically, the measurement frequency window was set between 10 and 1600 Hz, ensuring that the noise behavior and its dependence on device parameters could be effectively analyzed.

## Supplementary Information

Below is the link to the electronic supplementary material.

Supplementary Material 1.

## Data Availability

The data that support the findings of this study are available in the Supporting Information of this article.
