# Supplementary material for: Unraveling ionic switching dynamics in high-k dielectric double-gate transistors via low-frequency noise spectroscopy
Source: Nano Converg. 2025 Oct 3;12:48. doi: 10.1186/s40580-025-00512-2 (PMC12495002; doi:10.1186/s40580-025-00512-2)
Supplement: Supplementary file 1 — Supplementary Material 1. [file 40580_2025_512_MOESM1_ESM.docx]

Supporting Information

**Unraveling Ionic Switching Dynamics in High-*k* Dielectric Double-Gate Transistors via Low-Frequency Noise Spectroscopy**

*Soi Jeong^1^, Chang-Hyeon Han^2^, Been Kwak^2^, Ryun-Han Koo^3^, Youngchan Cho^4^, Jangsaeng Kim^5,6^, Jong-Ho Lee^7^, Daewoong Kwon^2^*, and Wonjun Shin^4^*,*

^1^Department of AI Semiconductor Engineering, Hanyang University, Seoul 04763, Republic of Korea

^2^Department of Electrical Engineering, Hanyang University, Seoul 04763, Republic of Korea

^3^Department of Electrical and Computer Engineering and Inter-university Semiconductor Research Center, Seoul National University, Seoul 08826, Republic of Korea

^4^Department of Semiconductor Convergence Engineering, Sungkyunkwan University, Suwon 16419, Republic of Korea

^5^Department of Electronic Engineering, Sogang University, Seoul 04107, Republic of Korea

^6^Department of System Semiconductor Engineering, Sogang University, Seoul 04107, Republic of Korea

^7^Department of Electrical and Computer Engineering, Seoul National University, Seoul 08826, Republic of Korea

S. Jeong, C. H. Han, and B. Kwak contributed equally to this work.

* E-mail: dw79kwon@hanyang.ac.kr, swj0107@skku.edu

**
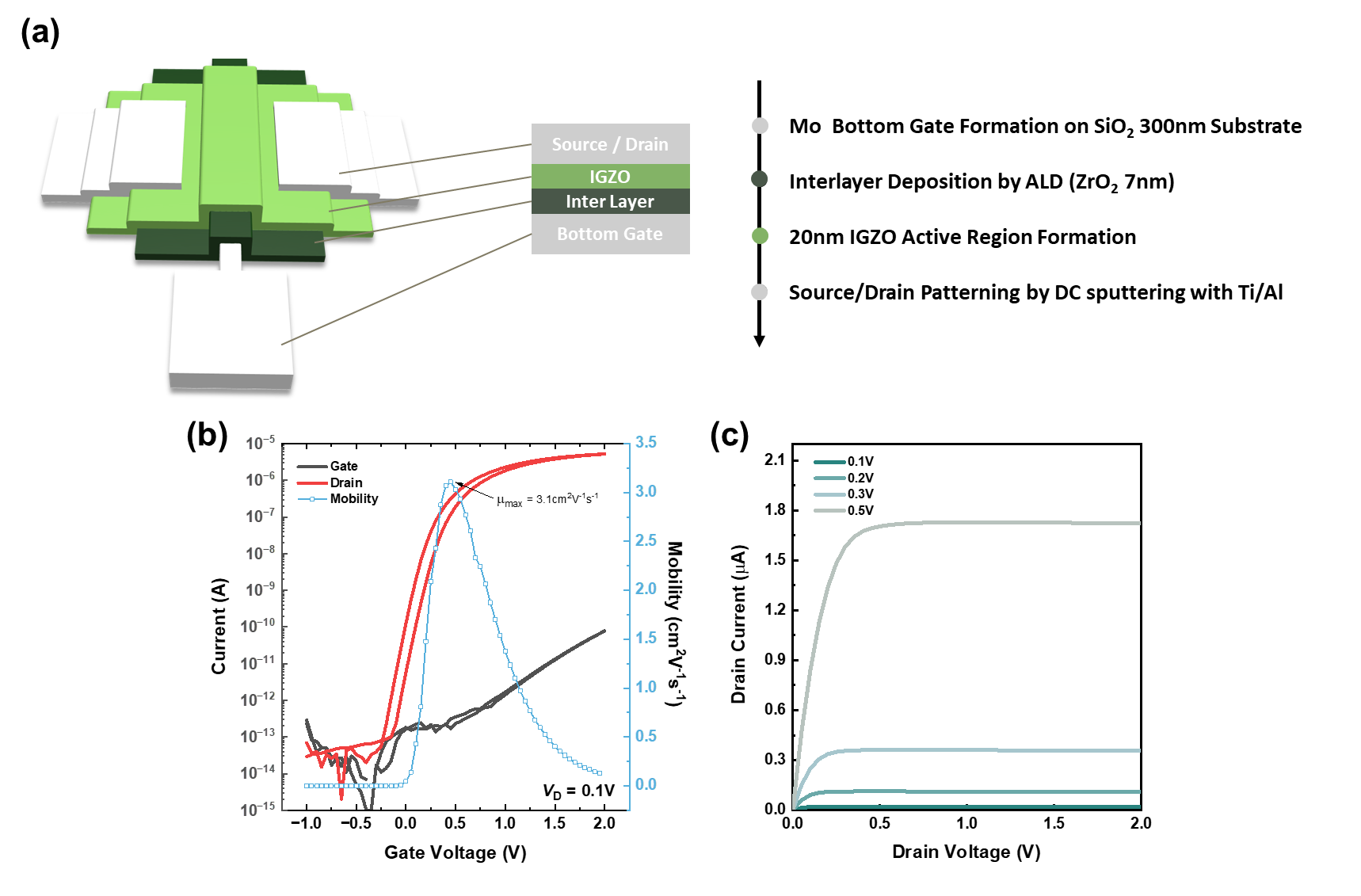
**

**Figure S1**. Fabricated single gate IGZO TFT and electrical characteristics used to evaluate the channel properties of the IGZO film. (a) Schematic and fabrication process flow of the single gate IGZO TFT, where a 20 nm-thick IGZO layer was deposited by RF sputtering, identical to the process described in the main manuscript. (b) Transfer curve measured under a low gate voltage range (left Y-axis, Y1) and the saturation mobility extracted from the curve (right Y-axis, Y2). A slight clockwise hysteresis is observed in the transfer curve, attributed to dielectric trap sites, distinct from the ferroelectric-like counterclockwise hysteresis discussed in the main manuscipt. (c) Output curve measured under gate voltages ranging from 0.1 V to 0.5 V.


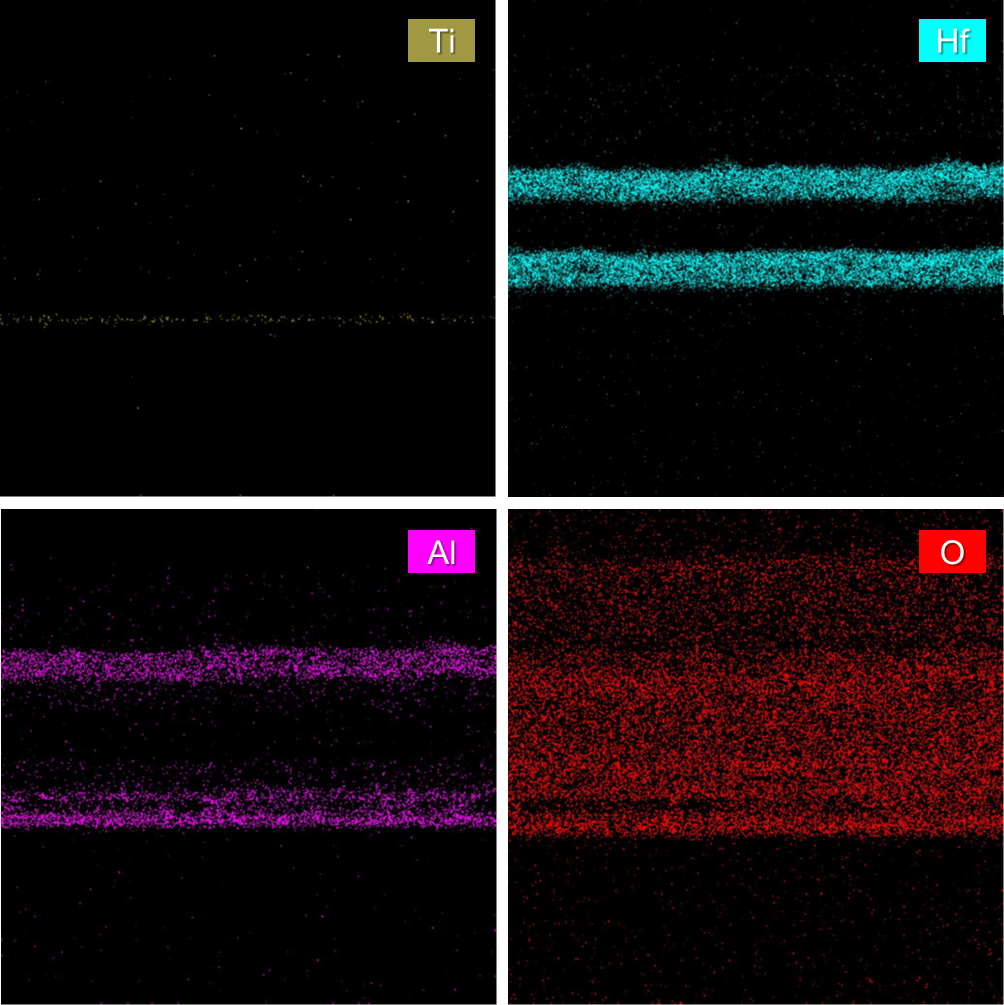


**Figure S2.** EDS analysis about dielectric elements of the stack of double gate IGZO TFT structure.


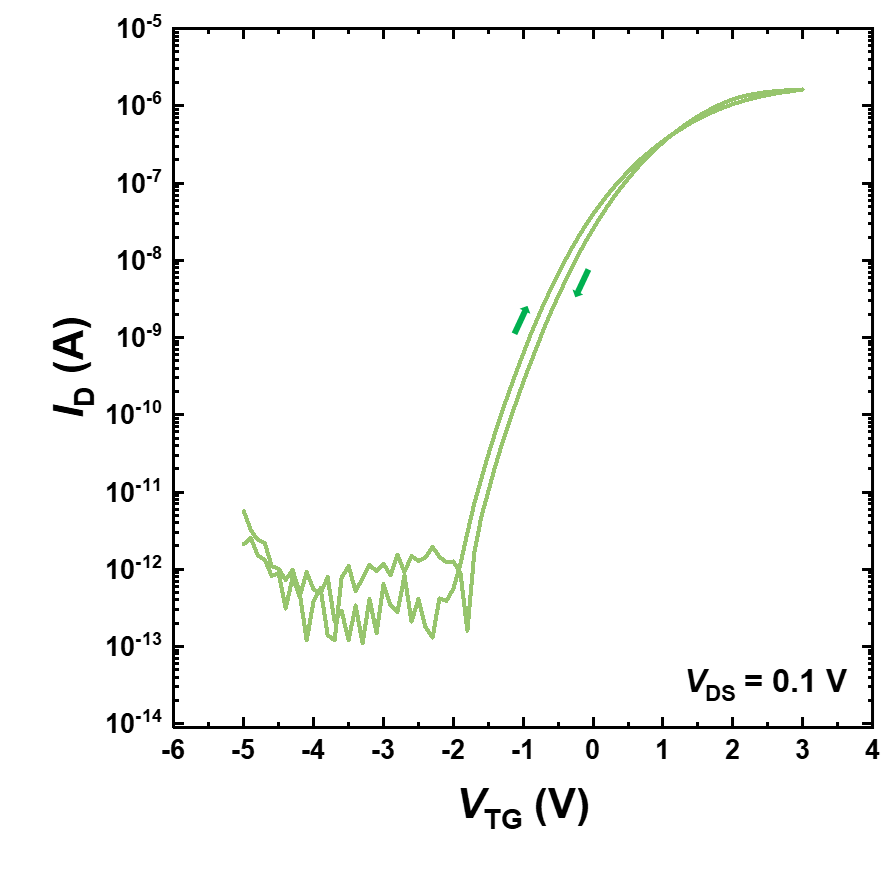


**Figure S3.** Top gate double sweep transfer curve (*I*_D_-*V*_TG_), exhibiting that the top gate TFT shows only a slight clockwise hysteresis, due to charge trapping within the dielectric layers in the wide sweep voltage range.


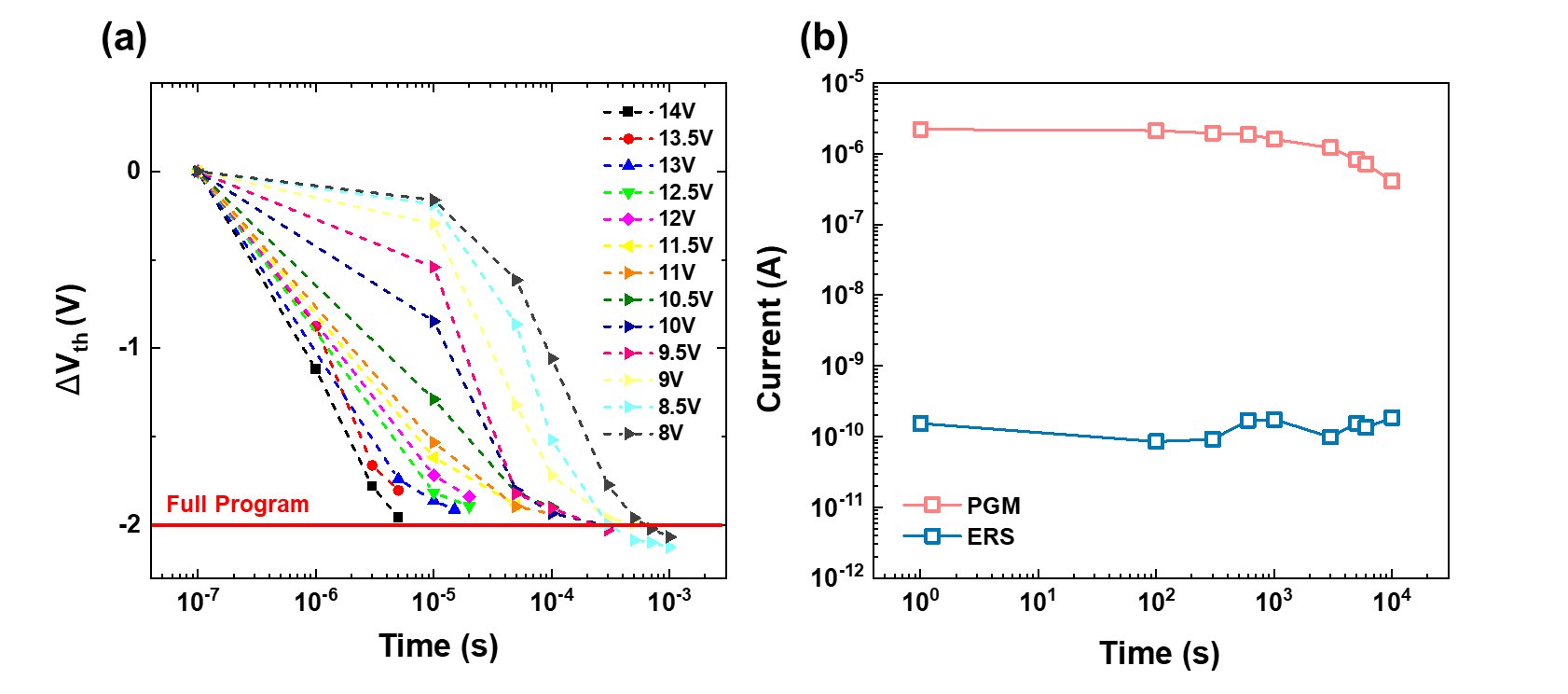


**Figure S4.** (a) Program speed characteristics under long activation conditions for ionic migration effect, demonstrating the time-dependent nature of the process. (b) Retention characteristics of the bottom gate TFT measured under fully programmed/erased conditions (8V, 1ms for PGM; –8V, 1ms for ERS).

**
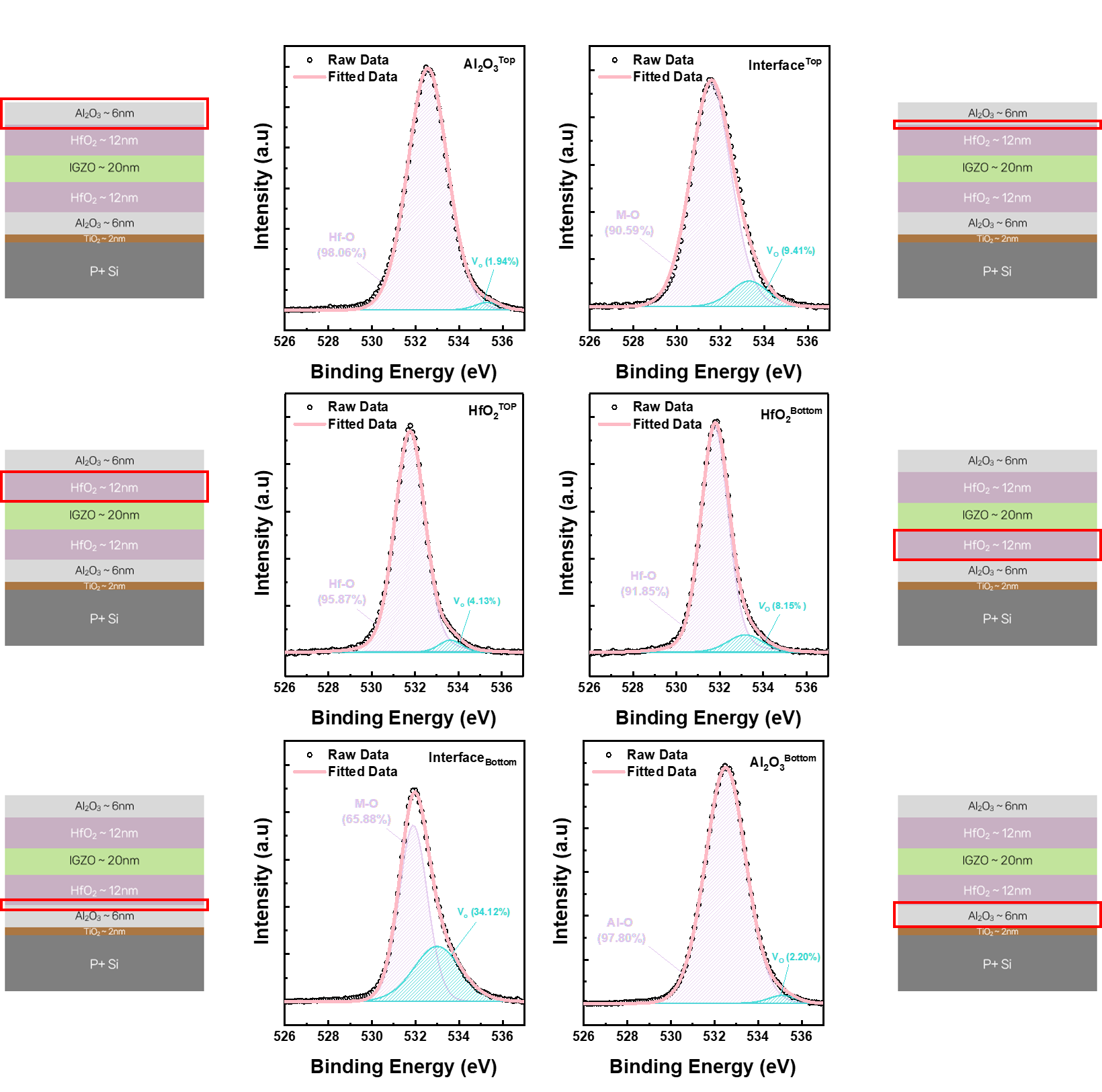
**

**Figure S5**. O1s XPS spectra of entire dielectric films collected from different positions in the device to compare the relative oxygen vacancy concentrations. Overall, a higher oxygen vacancy content is observed in the bottom stack.

**
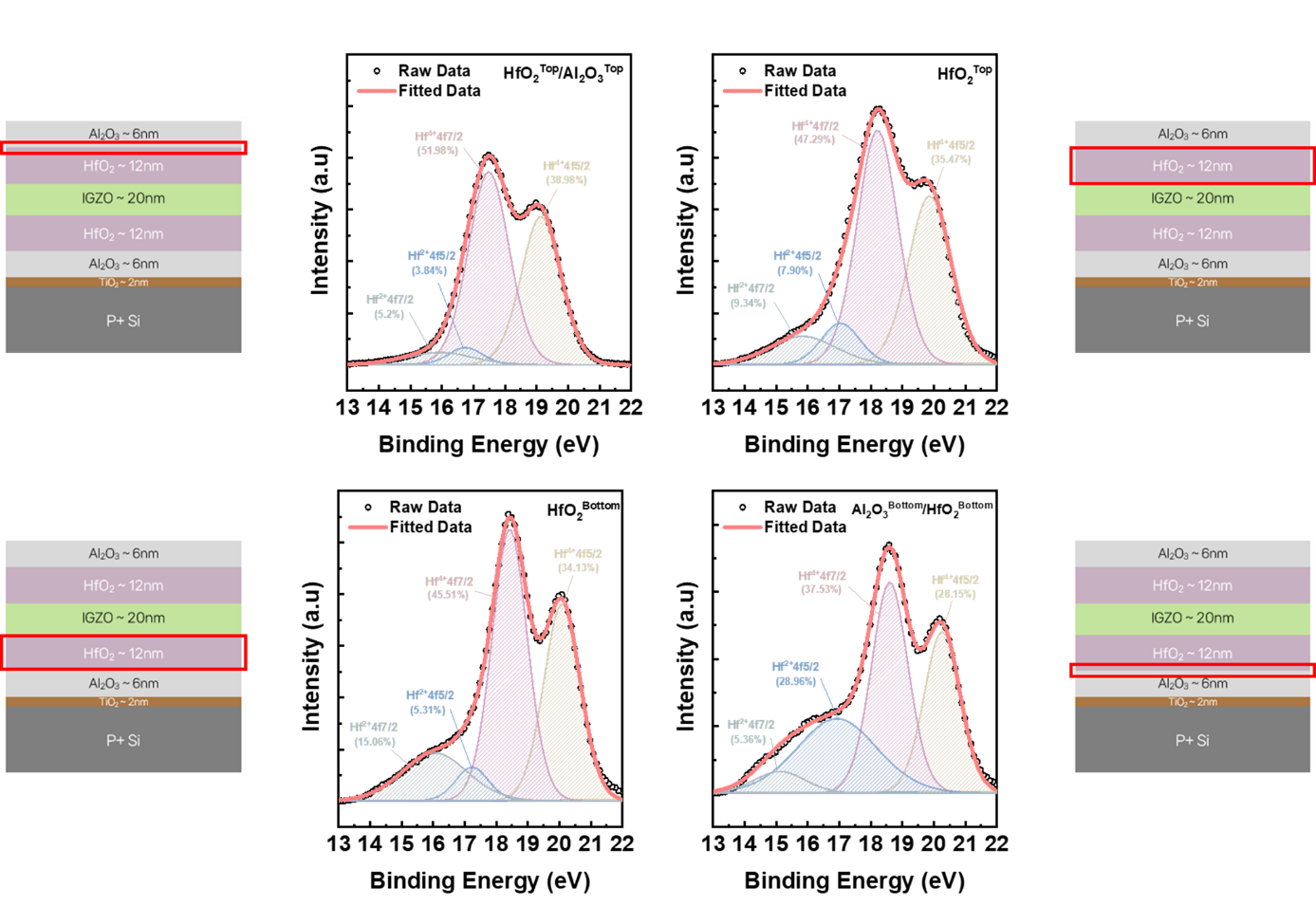
**

**Figure S6**. Hf4f XPS spectra of HfO_2_ films at various positions in the top and bottom gate stacks. Due to the presence of the TiO_2_ layer, the bottom HfO_2_ film consistently exhibits higher oxygen vacancy concentrations across all measured locations.


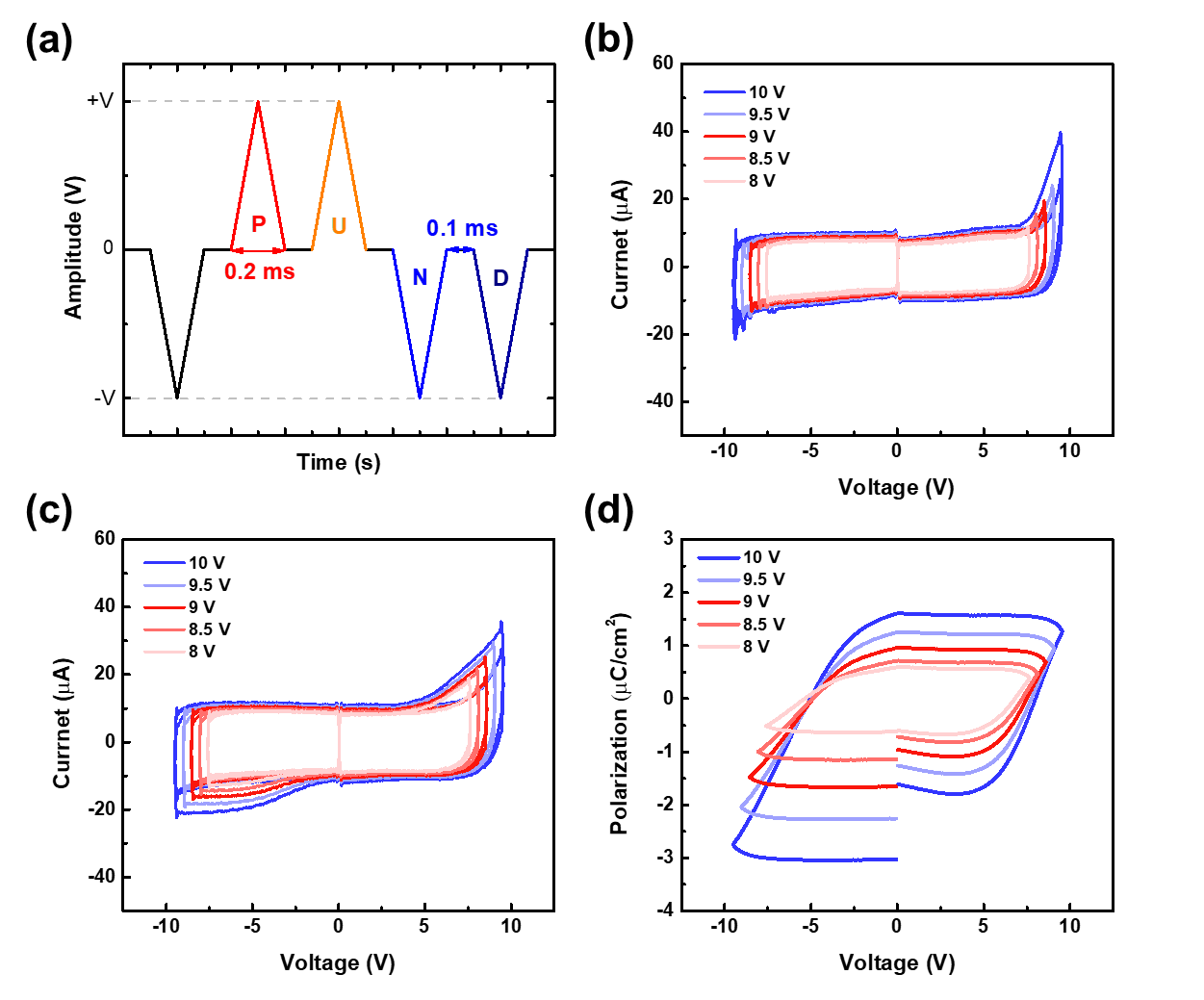


**Figure S7.** PUND measurements to verify the absence or presence of ferroelectric behavior in various dielectric stacks with Keithley 4200-SCS semiconductor parameter analyzer. (a) Schematic of applied pulse train. Polarization measurements were conducted within a voltage range of 8-10 V at 10 kHz. In this case, delay time between pulses is 0.1 ms. (b) *I*–*V* characteristics of HfO_2_/Al_2_O_3_ stacks, showing no observable switching current, indicating non-ferroelectric behavior. (c) *I*–*V* characteristics of TiO_2_/Al_2_O_3_/HfO_2_ stacks, showing a weak switching current attributed to oxygen vacancy-induced polarization effects. (d) Calculated polarization–voltage (*P*–*V*) curve derived from the switching current response of the TiO_2_/Al_2_O_3_/HfO_2_ stacks.


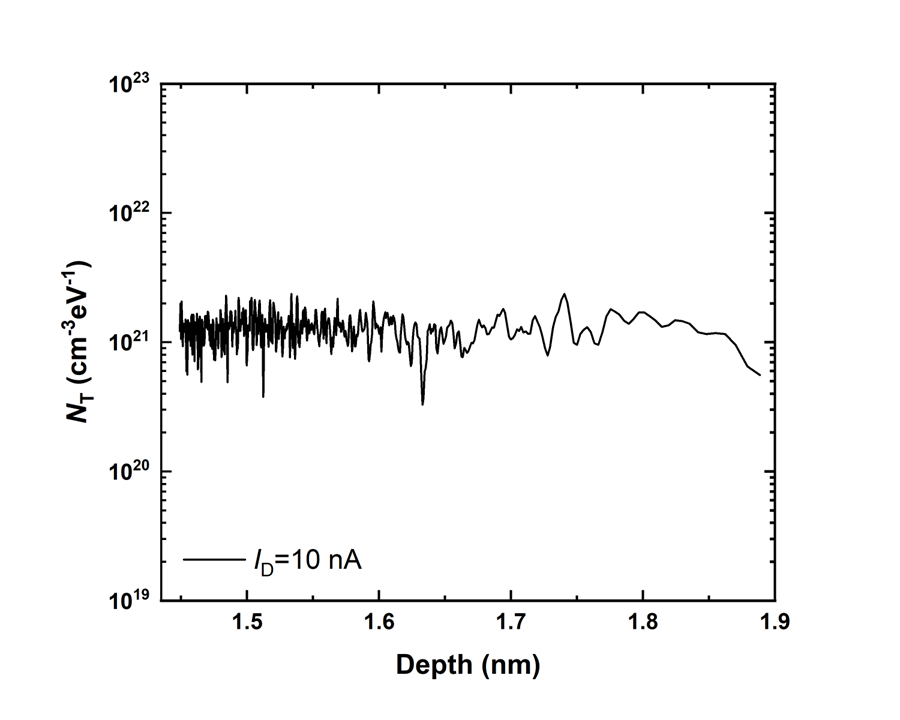


**Figure S8**. Top gate trap density profile derived from the noise spectrum at *I*_D_ = 10 nA, which is the highest measured current condition in the erase state.
